# Effects of Edible Insect Enrichment of Meat-Based and Meat Analogue Products on the Nutritional Composition, Techno-Functional Properties, and Acceptability to Consumers: A Systematic Review

**DOI:** 10.3390/foods15132329

**Published:** 2026-07-01

**Authors:** Isaac Amoah, Mauro Lombardo, Charles Diako, Comfort Adjei-Boamah, Vanessa Adu Sarpong, Elaine Rush

**Affiliations:** 1 Department of Biochemistry and Biotechnology, Kwame Nkrumah University of Science and Technology, Kumasi 00233, Ghana; cadjeiboamah1@st.knust.edu.gh (C.A.-B.); vadusarpong@st.knust.edu.gh (V.A.S.); 2 Department for the Promotion of Human Science and Quality of Life, San Raffaele Open University, Via di Val Cannuta, 247, 00166 Rome, Italy; mauro.lombardo@uniroma5.it; 3 School of Food Technology and Natural Sciences, Massey University, Auckland 0632, New Zealand; c.diako@massey.ac.nz; 4 Faculty of Health and Environmental Studies, Auckland University of Technology, Auckland 1142, New Zealand; elaine.rush@aut.ac.nz; 5 Riddet Centre of Research Excellence, Palmerston North 0632, New Zealand

**Keywords:** consumer acceptability, edible insects, insect protein, meat analogues, meat-based products, protein fortification, techno-functional properties

## Abstract

Background: Meat-based and meat analogue products are commonly consumed foods for protein nourishment across the globe. These food products are promising carrier media that can be targeted for the delivery of edible insect products as a strategy to overcome food neophobia. This systematic review aimed to evaluate the effect of edible insect enrichment on the nutritional, physicochemical, techno-functional, and acceptability of meat-based and meat analogue products for consumers. Methods: A systematic search for relevant articles was conducted on the databases PubMed, ScienceDirect, and Scopus on 5 November 2025. A total of 25 papers were included. Results: Edible insects and larvae from mealworms, crickets, grasshoppers, silkworm pupae, and superworms were commonly used to enrich meat-based and meat analogue products. Commonly reported meat products were sausages from pork and beef, pork patties, meat emulsion, and frankfurters. Soya flour was commonly used for meat analogue formulation. Conclusions: Acceptable edible insect incorporation is matrix-dependent, with conventional meat products generally tolerating low substitution levels (≈2.5–10%) before sensory and structural quality declines, whereas meat analogue systems can accommodate substantially higher inclusion (≈10–40% and, in some cases, up to ~60%). However, heterogeneity in formulations and sensory evaluation methods restricts direct comparison across studies.

## 1. Introduction

Protein is an essential component of living cells and the human diet. The global demand for protein, especially from sustainable sources, continues to increase [1]. Amino acids form the building blocks of proteins, and the nutritional quality of proteins in foods is dependent on the digestibility and bioavailability of these amino acids [2]. Dietary protein is largely sourced from plant and animal foods. Animal sources of proteins have a high biological value as they contain all the essential amino acids, are readily digestible, and contain essential minerals such as iron and zinc [2]. However, the production of animal-based proteins, particularly from livestock, comes with sustainability, environmental, and health-related challenges, which may be met by the production of alternative protein sources [3].

Plant-based sources of protein have gained increased research interest in the past decade as part of a broader strategy to promote their sustainable consumption. Despite not being intrinsically complete on their own in terms of their essential amino acid profiles, plant-based proteins, such as those from legumes, could be complemented with those from cereal grains as part of strategies to balance their amino acid profiles [4]. Plant-based proteins such as soybean, chia, and quinoa, however, have matched the essential amino acid profile of animal-based sources. Consequently, they are used in meat analogues or texturized plant-based “meat” products, which are acceptable to vegetarians and vegans [5], with their techno-functional properties and colour presentation traditionally made to imitate animal-based meat [6,7].

A recent systematic review of observational studies evaluated consumers’ perspectives toward the acceptance of plant-based meats [8]. The reduced carbon footprint, sustainability, and inclusion of plants in the daily healthy diet recommendations for Chinese people were reasons for choosing plant-based foods [9]. In a recent review, sensory attributes, including taste and appearance, were identified as the primary drivers for plant-based meat acceptance [10]. In that same review, the authors reported that meat analogues, when compared with beef and assessed for their sensory properties, had poor acceptance, particularly in taste and appearance [10]. The need to prioritize technological considerations and reformulation strategies that enhance taste and appearance, thereby increasing acceptance of plant-based meat, is therefore important.

Reformulating animal-based meat products and meat analogues through enrichment with powders from edible insects and their larvae is an innovative approach toward enhancing the nutrient density, promoting dietary diversity, and may enhance the organoleptic and textural attributes of meats and meat analogues [11,12]. Additionally, the strategy is also effective toward addressing neophobia tendencies associated with edible insect consumption [13,14] and is an important approach toward promoting the utilization of edible insects in new food product development [15,16]. Edible insects are rich sources of dietary fiber in the form of chitin, proteins, and fatty acids, and their composition varies according to the diet of the insect [17].

During the reformulation of meat-based products and meat analogues, the interaction between amino acids and fatty acids from these different food sources may affect the nutritional composition, techno-functional properties, and sensory attributes of the enriched products. It is therefore important to better understand the impact of edible insect powder enrichment on meat, meat-based products, and meat analogues to inform the most acceptable substitution. For instance, enrichment of pork sausages with mealworm larvae (*Tenebrio molitor*) and silkworm pupae (*Bombyx mori*) flours has been shown to improve nutritional value and physicochemical properties compared to non-enriched controls [18]. Similar benefits were observed when cricket flour was incorporated into meat emulsions [19], pork patties [20], and beef burgers [21], yielding products with superior functional and compositional attributes. Conversely, the incorporation of whole insect larvae powders from yellow mealworms and black soldier flies into pork and beef formulations was associated with increased cooking losses and deterioration in sensory and physicochemical qualities, particularly at higher inclusion levels [22]. These findings underscore the importance of careful evaluation of insect species, processing methods, and inclusion rates to ensure that enrichment strategies deliver nutritional and technological advantages without compromising consumer acceptance.

**Figure 1:**
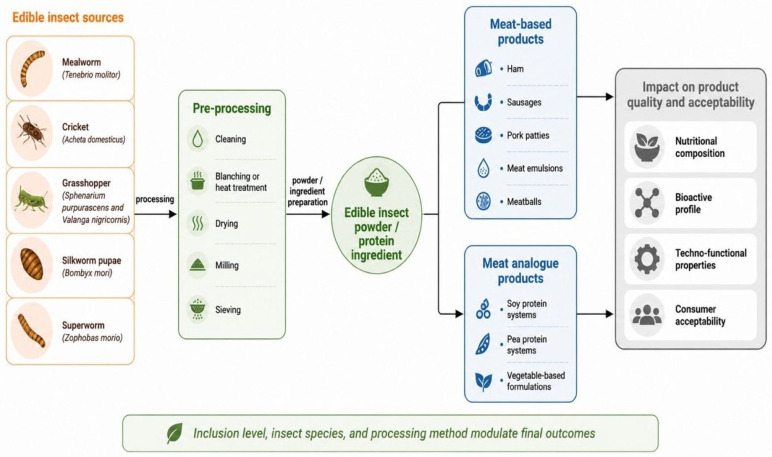
Conceptual framework illustrating the potential of edible insect enrichment on the nutritional composition, techno-functional properties, and consumer acceptability of meat-based and meat analogue products.

To provide a visual overview of the review scope, Figure 1 illustrates the potential relationships between edible insect processing, product formulation, and the nutritional, techno-functional, and consumer acceptability outcomes evaluated in meat-based and meat analogue products. The framework was developed from evidence reported in the broader literature and serves as a conceptual guide to the review. Prior to incorporation, edible insects such as superworms, mealworms, crickets, larvae, and grasshoppers undergo several processing steps, including cleaning, blanching or heat treatment, drying, milling, and sieving to obtain stable powdered forms suitable for food formulation. These edible insects are recognized for their high-quality protein, essential amino acids, healthy fatty acids, vitamins, and mineral compounds. The insect powders may then be incorporated into meat-based products such as ham, pork, sausages, meatballs, and meat emulsions, as well as meat analogue products developed from soy protein, pea protein, and vegetable-based systems. Edible insect enrichment has the potential to improve protein quality, micronutrient composition, water holding capacity, emulsification, texture, and overall product functionality. However, consumer acceptability remains dependent on sensory attributes such as flavour, colour, appearance, and texture, which may vary with insect species, processing methods, and quantity (or concentration) of the edible insect powder. Overall, the framework demonstrates the potential of edible insects as sustainable and functional ingredients for the development of nutritious and consumer-acceptable meat and meat analogue products.

Currently, reviews on the effects of edible insect powder enrichment of commonly consumed staple foods such as bakery, pasta, and noodle products are available [23,24,25]. However, systematic reviews on the effects of edible insect powder enrichment of meat and meat-based products and meat analogues on the nutritional, techno-functional, and acceptability of the enriched products are currently lacking. Previous studies have examined edible insects as alternative protein sources, focusing on their nutritional value, sustainability, consumer perceptions, and potential applications in food systems [23,26,27]. Nevertheless, they have generally been narrative in nature and have not systematically synthesized evidence specifically on the incorporation of edible insects into meat-based and meat analogue products. As a result, the extent to which edible insect enrichment influences nutritional composition, techno-functional characteristics, and consumer acceptability across different product categories remains unclear. By employing a systematic and reproducible search, screening, and study selection process, the present review provides a focused synthesis of the available evidence, identifies areas of agreement and inconsistency across studies, and highlights research gaps requiring further investigation. Therefore, this systematic review aims to evaluate the effect of edible insect enrichment on the nutritional, physicochemical, techno-functional, and acceptability of meat and meat-based products to consumers. Accordingly, this review sought to answer the following research question: Among meat-based and meat analogue products, how does enrichment with edible insect and larvae powders, compared with non-enriched control formulations, influence nutritional composition, techno-functional properties, and consumer acceptability?

## 2. Methods

**Figure 2:**
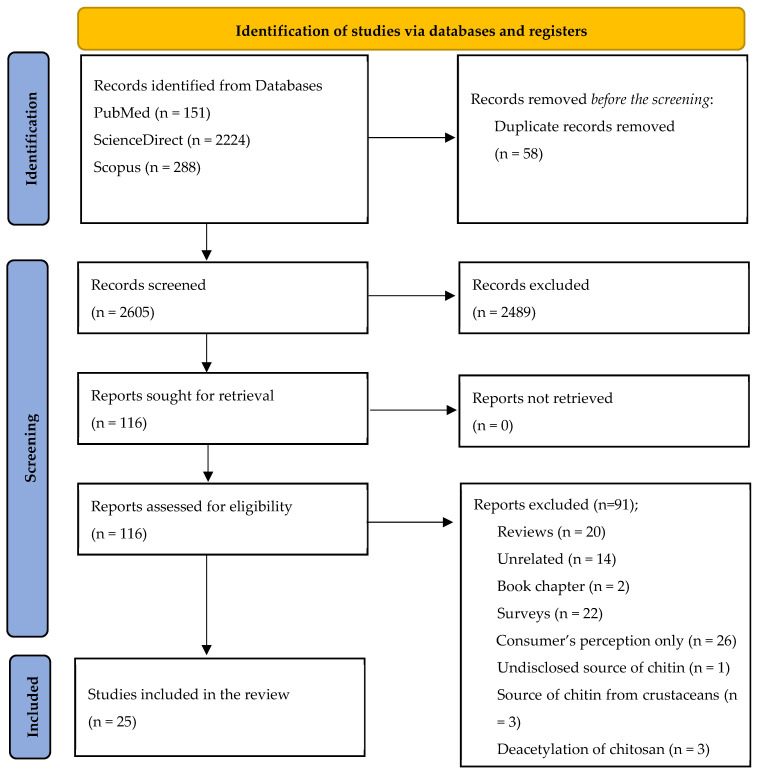
Flowchart summarizing studies evaluated and selected for the systematic review.

This systematic review was conducted in accordance with the guidelines of the updated Preferred Reporting Items for Systematic Reviews and Meta-Analysis (PRISMA) statement [28] (Figure 2, Supplementary Materials Table S1). A systematic search for articles was conducted on the databases PubMed, Scopus, and ScienceDirect on 5 November 2025.

The search term employed for PubMed was (edible insect* OR entomophagy) AND (meat product* OR meat burger OR sausage* OR meat patty* OR meat analogue OR meat-based dual protein food* OR texturized plant-based meat OR unconventional meat product* OR meat-based hybrid* OR meat substitute). For Scopus and ScienceDirect, the search term employed was (edible insect OR entomophagy) AND (meat product OR meat burger OR sausage OR meat analogue OR texturized plant-based meat OR unconventional meat product OR meat substitute). No filters or restrictions were used during the article searches on PubMed and Scopus, but the search on ScienceDirect was restricted to research articles to improve the relevance of the retrieved records and manage the database output. Additionally, due to limitations imposed by ScienceDirect on the length and use of wildcard operators such as (*), a simplified search syntax was employed while retaining the key concepts of edible insects, meat products, and meat analogues. These database-specific modifications resulted in differences in the number of records retrieved across databases.

Records obtained from PubMed, Scopus, and ScienceDirect were imported to the reference manager, ENDNOTE™ Library. Duplicate records were removed. The inclusion criteria adopted for the selection of articles for this systematic review included (i) articles must be original research; (ii) articles must be written in English with their full text readily accessible; and (iii) articles must report on meat or meat analogue products formulated with edible insects. The exclusion criteria were (i) studies that were published as review articles, book chapters, commentaries, letters to editors, surveys, and conference proceedings; (ii) studies that reported only on consumer perception or nutritional composition; and (iii) studies that did not disclose the source of chitin or obtained chitin from crustaceans or deacetylation.

Three independent authors, C.A-B., V.A.S., and I.A., critically extracted all the data, checked, and selected the articles for inclusion. In instances of disagreement among the authors, a consensus was reached through discussions with authors I.A. and C.D. The articles were exported to the reference manager ENDNOTE™ Library.

Study characteristics extracted included country of study, year of publication, type of insect used, pre-treatment method used for insect processing, form of edible insects used for the meat or meat analogue enrichment, substitution levels of the edible insects used for enrichment, type of meat or meat analogue product, study objectives, and study outcome.

The methodological quality of the included studies was evaluated using an adapted critical appraisal tool designed for experimental food formulation studies [29]. The assessment comprised nine domains: (i) clarity of study aims, which evaluated whether the research objectives were explicitly stated; (ii) appropriateness of study design, which assessed whether the experimental approach was suitable for addressing the stated objectives; (iii) adequacy of methods reporting, which examined the transparency and completeness of methodological descriptions; (iv) appropriateness of measurement tools, which assessed whether validated analytical and instrumental methods were used; (v) formulation description, which evaluated the extent to which ingredient composition, substitution levels, and processing procedures were reported; (vi) replication, which assessed whether sufficient analytical or experimental replication was described; (vii) outcome reporting, which examined the completeness of results presentation; (viii) statistical analysis, which evaluated the appropriateness and reporting of statistical procedures; and (ix) support of conclusions, which assessed whether the conclusions were consistent with the reported findings. Each domain was scored as either “Yes” (criterion met) or “No” (criterion not met), yielding a maximum score of 9 points. Studies scoring 7–9 points were classified as high quality, 4–6 points as moderate quality, and 0–3 points as low quality.

## 3. Results

### 3.1. Study Selection

A total of 2663 records were retrieved from the databases PubMed *(n* = 151), Scopus (*n* = 288), and ScienceDirect (*n* = 2224). The removal of duplicates (*n* = 58) from the retrieved articles (*n* = 2663) yielded 2605 studies. Following the screening of titles and abstracts, 2489 articles were excluded, resulting in 116 articles that were sought for full-text eligibility. A total of 91 articles were excluded, resulting in the final selection of 25 studies. A detailed description of the search and study selection process is summarized in Figure 2.

### 3.2. Geographical Distribution of Selected Studies

Most of the studies included in this systematic review (Table 1 and Table 2) originated from South Korea (*n* = 6), followed by Mexico (*n* = 3). China, Spain, the United States, Bulgaria, and Thailand each contributed two studies, while Brazil, Belgium, Portugal, India, Poland, and Indonesia each recorded one study. Geographically, Asia accounted for the highest number of publications (*n* = 12), followed by Europe (*n* = 7), North America (*n* = 5), and South America (*n* = 1). No studies were identified from Africa or Oceania. This is not surprising as Asian countries, particularly those of the Southeast region, are traditional consumers of edible insects [30]. Several studies that have investigated consumers’ attitudes toward the consumption of edible insects have reported higher tendencies of apparent food disgust or food neophobia in consumers from higher-income continents such as Europe and America, compared to their counterparts from Asia and Africa [31,32]. These two continents have traditionally been exposed to the consumption of edible insects; thus, the phenomenon remains common.

However, even though consumers in Europe and other high-income regions have indicated a dislike of edible insect consumption, increased interest in consuming food products enriched with insect flours and larvae-based protein extracts was reported [13,14]. Consequently, it was not surprising that Europe recorded the second-largest number of publications in this review. That is a testament to innovations in product development, particularly with a focus on meat and meat analogues in Europe. China, being the second Asian country reporting a higher number of publications, could be attributed to the fact that China is one of the highest pork-consuming countries in the world [33].

**Table 1:** Effects of edible insect powder enrichment in meat-based products and meat analogues on the nutritional composition.

Authors, Year	Country of Study	Edible Insects/ Larvae Used/Form Used	Pre-Treatment Method Used	Meat/Meat Analogue Product	Substitution Level	Findings
Albuquerque et al. [21]	Brazil	Cricket powder	Boiling and stove drying	Beef burger	0%, 8.25%, 12.5%, and 25%	↑ protein and ↓ fat content for beef burger enriched with 8.25% cricket and 8.25% soy protein compared to the control
Choi et al. [34]	South Korea	Powder from larvae of *Tenebrio molitor*, *Protaetia brevitarsis seulensis*, *Allomyrina dichotoma*, and *Gryllus bimaculatus*	Freeze-drying	Pork patties	20% *Tenebrio molitor*, *Protaetia brevitarsis seulensis*, *Allomyrina dichotoma*, and *Gryllus bimaculatus* larvae powder-enriched pork patties	↑ protein content for all the enriched patties, except the *Allomyrina dichotoma* enriched pork patties, compared to the control. ↓ fat for all the enriched pork patties compared to the controls
Cruz-López, Escalona-Buendía, et al. [35]	Mexico	Grasshopper (*Sphenarium purpurascens*) (alkaline-extracted and alkalization + ultrasound-extracted proteins)	Seasoning application, roasting, and refrigeration	Sausages	2.5%, 5%, and 7.5%	↑ total protein content
Kim et al. [18]	USA	Mealworm larvae and silkworm pupae powder	Freeze-drying, grinding, defatting, and hydrolysis	Sausages	10%	↑ protein and ↓ fat in all formulations compared to the control. ↑ total fiber in defatted and ↓ total fiber in defatted and acid hydrolyzed formulations of both mealworm larvae and silkworm pupae compared to the control
Park et al. [36]	South Korea	Silkworm pupae (*Bombyx mori*) powder	Vacuum drying	Meat batters	Only (5%, 10%, and 15%) with or without 1% transglutaminase	↑ protein and ↑ fat content in the formulated samples
Rodríguez-Párraga et al. [37]	Spain	*Tenebrio molitor* (powder (defatted) and whole)	Defatting (Supercritical fluid extraction)	Bologna-type sausages	7.5% and 15% whole *Tenebrio molitor* powder 7.5% and 15% defatted *Tenebrio molitor* powder	↑ protein, ↑ total fiber, ↓ fat, ↑ calcium, ↑ potassium, and ↑ magnesium content with increasing edible insect substitution
Zhang et al. [38]	China	Mealworm larvae	Freeze-drying and microwave-drying	Frankfurters (post-rigor lean pork meat and pork fat)	5%, 10%, and 15%	↑ release of fat from emulsification of the frankfurters with increasing the freeze- dried edible insect flour substitution. No significant difference between the protein and fat content of the various formulations and the control.
Scholliers et al. [39]	Belgium	Superworm (*Zophobas morio*) larvae paste	Mincing	Hybrid cooked sausage	0%, 5%, 10%, 25%, and 50%	↑ fat and ↑ protein content compared to the control
Zhang et al. [40]	China	*Tenebrio molitor* larvae protein	Freezing with liquid nitrogen	Frankfurters (lean pork and pork back fat)	5%, 10%, 15%, 20%, and 25%	↓ fat content and ↑ release of fat from emulsification of the frankfurters with increasing edible insect substitution
Kim et al. [19]	USA	Cricket (*Acheta domesticus*) powder	NR	Meat emulsion	5% and 10%	↑ protein content, ↑ acid detergent fiber, ↑ phosphorus, ↑ potassium, ↑ magnesium, ↑ zinc, and ↑ manganese in all formulations compared to the control
Carvalho et al. [41]	Portugal	*Tenebrio molitor* and *Alphitobius* powder	NR	Pork-based hybrid hams	10% *Tenebrio molitor* powder, 10% *Alphitobius* powder and 5% *Tenebrio molitor* powder, and 5% *Alphitobius* powder	↑ protein, ↑ lipid, and ↑ total phenolic content in the 10% *Tenebrio molitor* powder-enriched hams. ↑ sodium, ↑ calcium, ↑ potassium, and ↑ magnesium content compared to the control
Hospital et al. [42]	Spain	Mealworm (*Tenebrio molitor*) powder	NR	Dry fermented sausage (salchichón)	0%, 5%, 10%, and 15%	↑ protein, ↑ fiber, and ↓ fat content for the enriched samples
Kolev et al. [43]	Bulgaria	House cricket (*Acheta domesticus*) and yellow mealworm (*Tenebrio molitor*) powder	NR	Sausages	2% of each in separate samples	↑ protein and ↑ fat content in all formulations compared to the control
Singh et al. [44]	India	Locust protein hydrolysates	Defatting, ultrasonication, and microwave heating	Meat emulsion (mutton emulsion)	1.5% hydrolysate and 1.5% hydrolysate pre-processed with microwave or ultrasonication for (0, 7, and 14 days of storage)	↓ thiobarbituric acid reactive substances, ↓ free fatty acids, and ↓ total carbonyl content in formulations enriched with 1.5% of the locust protein hydrolysates on days 7 and 14 of storage
Yong et al. [45]	South Korea	(*Tenebrio molitor*, *Protaetia brevitarsis*, and *Allomyrina dichotoma*) larvae extract	Freeze-drying	Tteokgalbi (pork ham and pork back fat)	1% and 2%	↑ total phenolic content in *Protaetia brevitarsis*-enriched products and ↑ reducing power with increasing edible insect substitution
Boonarsa et al. [46]	Thailand	Silkworm pupae powder	Hot air oven drying	Silkworm meat analogue	0%, 5%, 10%, and 15%	↑ protein, ↑ amino acid, ↑ fiber, ↑ lipid, ↑ total phenolic, and ↑ total flavonoid content for the enriched products with increasing edible insect substitution
Kim et al. [47]	South Korea	Mealworm powder	Freezing and thawing	Jerky analogue	0%, 20%, 40%, 60%, 80%, and 100%	↑ histidine, ↑ isoleucine, ↑ leucine, ↑ threonine, ↑ valine, and ↓ methionine and ↓ phenylalanine amino acid concentration with increasing edible insect substitution
Momchilova et al. [48]	Bulgaria	Cricket powder	NR	Pork meatballs	0, 3.33, 5, and 10 g/kg^−1^	↑ protein and ↑ dietary fiber in formulated samples
Kim et al. [49]	South Korea	*Tenebrio molitor* larvae powder	Freezing and thawing	Texturized vegetable protein	0%, 25%, 50%, 75%, and 100%	↓ fat exudate for the formulation containing 50% texturized vegetable protein plus 50% *Tenebrio molitor* larvae with transglutaminase, compared with the other formulations
Chao et al. [50]	South Korea	Mealworm powder	NR	Meat analogue extrudates	0%, 15%, and 30%	↑ fibrousness of extrudate
Cruz-López, Álvarez-Cisneros, et al. [51]	Mexico	Grasshopper (*Sphenarium purpurascens*) powder	NR	Sausage	0%, 3%, 5%, 7%, and 10%	↑ protein and ↑ fat content, for all formulations compared to the control
Bottle et al. [52]	Mexico	Cricket powder	NR	Textured vegetable-insect proteins (TVIPs) containing soy flour, soy protein concentrate, and pea protein concentrate	0%, 10%, and 20%	↓ protein (soy protein concentrate), ↑ dietary fiber (soy protein concentrate), and ↑ fat content (all formulations) for the enriched textured vegetable-insect proteins
Krawczyk et al. [53]	Poland	Mealworms (*Alphitobius diaperinus*)	NR	Burger analogue	0% and 21.6%	↓ protein and ↑ total lipid content with increasing edible insect substitution
Nakagawa et al. [54]	Thailand	Cricket (*Acheta domesticus*) powder	NR	Meat analogue	5%, 6%, and 7%	↑ soluble protein content of the cricket powder-enriched sample
Priyatnasari et al. [55]	Indonesia	Javanese grasshopper (*Valanga nigricornis*) fillet	NR	Meat analogue	0%, 10%, 20%, and 30%	↑ protein content with increasing edible insect substitution

Keywords: NR—not reported; ↑—high/increased; ↓—low.

**Table 2:** Effects of edible insect powder enrichment in meat-based products and meat analogues on the techno-functional properties and acceptability.

Authors, Year	Country of Study	Edible Insects and Their Larvae/Form Used for Enrichment	Pre-Treatment Method Used	Meat or Meat Analogue Product	Substitution Levels	Acceptable Substitution	Sensory Scores	Findings
Albuquerque et al. [21]	Brazil	Cricket powder	Boiling and stove drying	Beef burger	8.25%, 12.5%, and 25%	12.5% cricket flour with 0% soy protein	NR	↑ hardness, ↑ gumminess, ↑ chewiness, ↓ cohesiveness, ↓ springiness, and ↓ L* for 12.5% cricket-enriched beef burger compared to the control
Choi et al. [34]	Korea	Powder from larvae of *Tenebrio molitor*, *Protaetia brevitarsis seulensis*, *Allomyrina dichotoma*, and *Gryllus bimaculatus*	Freeze-drying	Pork patties	20% for each edible insect type	20% (*Gryllus bimaculatus*)	Using 10 trained panelists, the formulation containing 20% substitution had an overall preference of 2.60, compared to 4.30 and 4.20 for the positive and negative controls, respectively	↑ pH and ↑ water holding capacity in all formulated products. ↑ hardness, ↑ cohesiveness, ↑ chewiness, and ↑ gumminess in the *Gryllus bimaculatus*-enriched products compared to the controls
Cruz-López, Escalona-Buendía, et al. [35]	Mexico	Grasshopper (*Sphenarium purpurascens*)	Seasoning, roasting, refrigeration, alkaline-only, and alkalization-ultrasound extraction of grasshopper protein	Sausage	2.5%, 5%, and 7.5%	5% (alkalization-ultrasound-assisted extraction)	Using 100 consumers, the control was preferred by 42 consumers and had the highest percentage of 43.75%, and 40 consumers with a slightly lower percentage of 41.66% favoured the 5% (alkalization-ultrasound-assisted extraction) sample	↑ pH, ↓ L*, ↑ b*, ↓ firmness, and ↓ hardness in all formulations compared to the control
Kim et al. [18]	USA	Mealworm larvae and silkworm pupae powder	Freeze drying, grinding, defatting, and hydrolysis	Sausage	10%	10% defatted mealworm larvae and 10% defatted silkworm pupae flour	NR	↑ pH, ↑ L*, ↑ cohesiveness, ↑ gumminess, ↑ chewiness in defatted and ↓ pH, ↓ L*, ↓ cohesiveness, ↓ gumminess, and ↓ chewiness in defatted and acid hydrolyzed formulations of both mealworm larvae and silkworm pupae compared to the control. ↑ hardness in all formulations compared to the control
Park et al. [36]	South Korea	Silkworm pupae (*Bombyx mori*) powder	Vacuum drying	Meat batters	5%, 10%, and 15% only and 5%, 10%, and 15% + 1% transglutaminase	15% silkworm pupae with 1% transglutaminase	NR	↑ pH, ↑ hardness, ↑ gumminess, and ↑ chewiness with all formulations compared to the control
Rodríguez-Párraga et al. [37]	Spain	*Tenebrio molitor* powder	Defatting using supercritical fluid extraction	Bologna-type sausage	7.5% and 15% whole *Tenebrio molitor* powder 7.5% and 15% defatted *Tenebrio molitor* powder	7.5% and 15% defatted *Tenebrio molitor* powder	Using 50 panelists, the overall acceptance of all sausages ranged from 6.91(control) to 5.61, except in the formulation with 15% whole *Tenebrio molitor* powder, which scored 3.93	↑ hardness and ↓ L* with increasing edible insect substitution
Zhang et al. [38]	China	Mealworm larvae powder	Freeze-drying and microwave drying	Frankfurters (post-rigor lean pork meat and pork fat)	0%, 5%, 10%, and 15% freeze-dried mealworm larvae powder 0%, 5%, 10%, and 15% microwave-dried mealworm larvae powder	10% incorporation of microwave-dried mealworm larvae powder	Using a 16-member sensory panel, the control group had the highest scores for interior colour, uniformity, flavour, and juiciness; however, the score for the substitution with 5% microwave-dried mealworm larvae powder was not significantly different from the control (*p* > 0.05)	↓ hardness, ↓ resilience, ↓ springiness, ↓ chewiness, ↓ adhesiveness, ↓ fracturability, and ↓ L*, with increasing freeze-dried larvae
Zhang et al. [40]	China	*Tenebrio molitor* larvae powder	Liquid nitrogen freezing	Frankfurters (lean pork and pork back fat)	5%, 10%, 15%, 20%, and 25%	5% and 10%	Using a 16-member sensory panel, except for flavour, the scores for all other sensory attributes gradually decreased with increasing edible insect substitution.	↓ L*, ↓ hardness, ↓ resilience, ↓ springiness, ↓ fracturability, ↓ chewiness, and ↓ tightness with increasing edible insect substitution
Hospital et al. [42]	Spain	Mealworm (*Tenebrio molitor*) powder	NR	Dry fermented sausage (salchichón)	0%, 5%, 10%, and 15%	5%	Using 18 panelists familiar with the sensory characteristics of dry fermented meat products, replacing 10% or more pork meat with insect flour resulted in significant differences across all attributes	↑ chewiness and ↑ cohesiveness with increasing edible insect substitution. ↓ hardness, ↓ L*, and ↑ pH in all formulations compared to the control
Kim et al. [19]	USA	Cricket (*Acheta domesticus*) powder	NR	Meat emulsion	5% and 10%	10%	NR	↑ hardness, ↑ gumminess, and ↑ chewiness in enriched formulations compared to the control
Kolev et al. [43]	Bulgaria	House cricket (*Acheta domesticus*) and yellow mealworm (*Tenebrio molitor*) powder	NR	Sausage	2%	2% house cricket powder	Using 5 trained panelists, both formulations had low scores for all the sensory attributes, but the sausages containing yellow mealworm flour received lower scores for appearance, texture, and flavour	↑ plasticity and ↑ structural strength after 7 days of storage
Singh et al. [44]	India	Locust protein hydrolysates	NR	Meat emulsion (mutton emulsion)	1.5% hydrolysate and 1.5% hydrolysate pre-processed with microwave or ultrasonication for (0, 7, and 14 days of storage)	1.5% hydrolysate	Using 10 trained panelist, mutton emulsion enriched with ultrasonication recorded the highest overall acceptability score of 6.6 on day 7, followed by 6.5 for the microwave pre-processed mutton emulsion, and then 6.4 for the untreated hydrolysates mutton emulsion compared to 6.2 for the control	↑ flavour, ↑ texture, ↑ colour, and ↑ appearance of the meat emulsion enriched with the locust protein hydrolysate on day 7
Yong et al. [45]	South Korea	(*Tenebrio molitor*, *Protaetia brevitarsis* and *Allomyrina dichotoma*) larvae extract	NR	Tteokgalbi (pork ham and pork back fat)	1% and 2%	2% *Allomyrina dichotoma*	NR	↓ brownness index and ↓ b* with increasing number of days of storage. ↑ cooking yield and ↓ shear force with increasing edible insect substitution
Cruz-López, Álvarez-Cisneros, et al. [51]	Mexico	Grasshopper (*Sphenarium purpurascens*) powder	NR	Sausage	0%, 3%, 5%, 7%, and 10%	7% and 10%	Using 10 consumers, the overall liking for formulations with 7 and 10% incorporated grasshoppers was 3.3 compared to 4.2 for the control	↑ hardness, ↑ springiness, ↑ gumminess, ↑ chewiness, and ↓ L* in all formulations compared to the control
Scholliers et al. [39]	Belgium	Superworm (*Zophobas morio*) larvae paste	Vacuum packing	Hybrid cooked sausage	0%, 5%, 10%, 25%, and 50%	25% and 50%	NR	↓ cooking loss in all formulations compared to the control at temperatures 70 °C and 80 °C
Boonarsa et al. [46]	Thailand	Silkworm pupae powder	Hot air oven drying	Silkworm meat analogue	5%, 10%, and 15%	15%	NR	↑ firmness, ↑ hardness, ↑ cohesiveness, ↓ adhesiveness, ↑ springiness, ↑ chewiness, ↓ L*, and ↓ water holding capacity with increasing edible insect substitution
Kim et al. [47]	South Korea	Mealworm powder	Thawing	Jerky analogue	0%, 20%, 40%, 60%, 80%, and 100%	40%	NR	↓ pH with increasing edible insect substitution. ↑ water activity in the control as compared to the formulations with the edible insect
Kim et al. [49]	South Korea	*Tenebrio molitor* larvae powder	Freezing and thawing	Texturized vegetable protein	0%, 25%, 50%, 75%, and 100%	50% texturized vegetable protein plus 50% *Tenebrio molitor* larvae with transglutaminase	NR	↓ pH and ↓ water holding capacity with increasing edible insect substitution. ↑ hardness, ↑ gumminess, and ↑ chewiness for the sample containing 50% texturized vegetable protein plus 50% *Tenebrio molitor* larvae with transglutaminase
Momchilova et al. [48]	Bulgaria	Cricket powder	NR	Pork meatballs	0, 3.33, 5, and 10 g/kg^−1^	10 g/kg^−1^	NR	↓ pH, ↑ L*, ↑hardness, and ↑ gumminess in the formulations that contained the edible insect compared with the sample made of only soy protein
Bottle et al. [52]	Mexico	Cricket powder	NR	Textured vegetable-insect proteins containing soy flour, soy protein concentrate, and pea protein concentrate	10% and 20%	20% (protein pea concentrate)	NR	↓ L*, ↑ hardness, and ↑ chewiness in pea and soy protein concentrates with increasing edible insect substitution
Carvalho et al. [41]	Portugal	*Tenebrio molitor* and *Alphitobius* powder	NR	Pork-based hybrid hams	10% for each edible insect and combined 5% of *Tenebrio molitor* and 5% *Alphitobius*	5% of *Tenebrio molitor* + 5% *Alphitobius* and 10% *A. diaperinus*	Using a 40-member untrained panel, the hybrid ham formulations with insect powders were well-received in terms of overall appearance, flavour, and texture, particularly the sample with 10% *A. diaperinus* and the combination of 5% *T. molitor* and 5% *A. diaperinus*	↑ firmness and ↑ adhesiveness in 10% *Alphitobius* powder, followed by 5% of *Tenebrio molitor* + 5% *Alphitobius* and then 10% *Tenebrio molitor*. Equal cohesiveness was observed in all formulations compared to the control
Chao et al. [50]	South Korea	Mealworm powder	NR	Meat analogue extrudates	0%, 15%, and 30%	30%	NR	↑ hardness, cohesiveness, and gumminess of extrudate containing the mealworm powder from temperature 120°C to 108°C. ↓ water holding capacity of the formulation containing 30% of the edible insect.
Krawczyk et al. [53]	Poland	Mealworms (*Alphitobius diaperinus*)	NR	Burger analogue	21.6%	21.6%	Using 24 panelists, the overall liking was highest for formulation B5 (5.88), followed by B10 (5.46) and B0 (5.25). The mean scores for all sensory attributes were approximately 5.0	↓ pH, ↓ L*, ↓ hardness, ↓ chewiness, and ↓ springiness with increasing edible insect substitution. ↑ cohesiveness and ↑ gumminess in the formulation containing 5% of the edible insect compared to the control.
Nakagawa et al. [54]	Thailand	Cricket (*Acheta domesticus*) powder	NR	Meat analogue	5%, 6%, and 7%	6%	NR	↑ water holding capacity, ↑ hardness ↓ cohesiveness, ↓ chewiness, ↓ springiness, and ↓ adhesiveness in all samples compared to the control
Priyatnasari et al. [55]	Indonesia	Javanese grasshopper (*Valanga nigricornis*) fillet	NR	Meat analogue	0%, 10%, 20%, and 30%	30%	Using 9 trained assessors, the preference score for the formulation with 30% edible insect substitution was 2.70 compared to 2.89 for the control	↓ L* and ↓ toughness with increasing edible insect substitution. ↓ hardness in all the formulations compared to the control

Keywords: NR—not reported; ↑—high/increased; ↓—low.

### 3.3. Publication Output Generated on the Application of Edible Insects in Meat-Based and Meat Analogue Products

**Figure 3:**
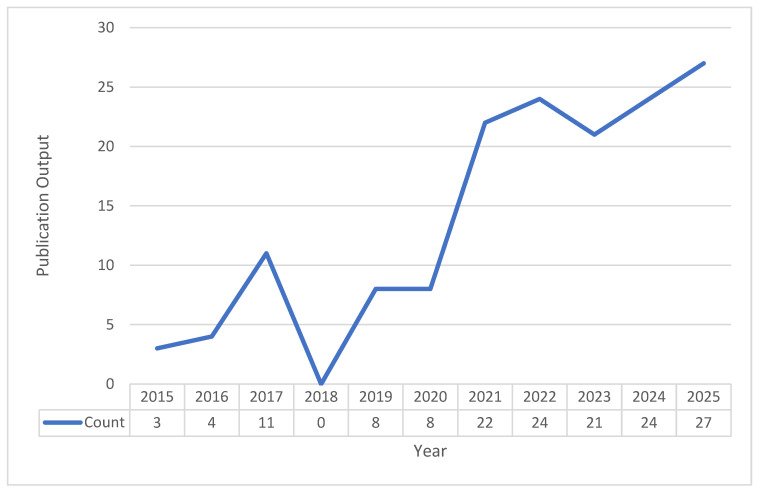
Trends in publication output showing the application of edible insects over 10 years from the PubMed database (2015–2025).

A systematic search for articles that sought to establish the trends in publication output with a focus on the application of edible insects over the past 10 years shows a gradual but marked increase in scientific attention to edible insects as alternative protein sources (Figure 3, Supplementary Materials Table S2). Publication output from the PubMed database increased from only 3 articles in 2015 to 27 articles in 2025, with the highest rate of increase occurring after 2020, reflecting increased global awareness of the need to change food production to address food security challenges, reduce environmental pressures associated with livestock production, and develop innovative protein-enriched food products. This trend highlights the emerging role of edible insects in the development of sustainable food and meat products.

### 3.4. Quality Assessment of Included Studies

Overall, the methodological quality of the included studies was high (Table 3). Quality scores ranged from 7/9 to 9/9, with all studies meeting the threshold for high quality. Four studies achieved the maximum score of 9/9, while the majority scored 8/9. Lower scores were primarily attributable to incomplete reporting of replication procedures or insufficient justification of conclusions relative to the reported findings. No study was classified as moderate or low quality, indicating a generally low risk of methodological bias among the included evidence base.

## 4. Discussion

### 4.1. Edible Insects Used for the Enrichment of Meat and Meat Analogue

This systematic review demonstrates that edible insects are promising functional ingredients for improving the nutritional quality of meat and meat analogue products. Across the included studies, insect incorporation affected techno-functional properties, such as texture, colour, water holding capacity, and cooking performance. The observed variability was largely influenced by four key factors. First, the insect species determined the nutritional composition, protein characteristics, and chitin levels, resulting in differences in the functional performance of the final products. Second, processing and pre-treatment methods, including drying, roasting, blanching, and milling, modified the physicochemical properties of insect ingredients, thereby influencing their functionality, sensory attributes, and stability. Third, the food matrix composition affected interactions between insect ingredients and other food components, leading to variations in product structure, texture, moisture retention, and overall quality. Finally, the level of insect substitution influenced the magnitude of nutritional enhancement and technological changes, with moderate inclusion levels generally maintaining acceptable product quality and consumer acceptability. Enrichment with edible insect powders generally increased crude protein content and dietary fiber; the protein increase reflects the amino acid-rich composition of insect flours relative to the displaced ingredient, while the fiber increase is attributable primarily to insect chitin, an indigestible polysaccharide not present in conventional meat [17]. For meat analogues, insect enrichment improved the amino acid profile as well as taste, addressing a common challenge for consumer acceptance. The substitution of meat and meat analogues with 5% edible insect and larvae powder resulted in products with high consumer acceptance. The textural attributes of the enriched products improved in hardness and cohesiveness.

### 4.2. Effect of Edible Insect Enrichment of Meat or Meat Analogue on Protein Content

In this review, enrichment of meat-based products and meat analogues increased protein and dietary fiber content, thereby improving the nutrient density of the enriched products. Generally, formulations with cricket showed protein contents ranging from 12.29–24.87% compared to 12.74–21.72% for their controls. However, the study by Bottle et al. [52] interestingly reported a substantially higher range of protein values from 54.6–71.4% for the control and 55.1–70.90% for the experimental samples. According to Albuquerque et al. [21], the beef burgers enriched with 8.25% cricket flour and 8.25% soy protein, as well as those enriched with 12.5% cricket flour and 12.5% soy protein, had a higher protein content than the control (made of meat alone) and other formulations with the cricket flour. There was no difference (*p* > 0.05) in protein content between the beef burger with 25% cricket flour and the control of 100% meat. Similar observations have been reported in the broader literature, where edible insect ingredients have been shown to increase the protein content of reformulated food products. A study conducted by Gomes Martins, et al. [56], for example, formulated beef patties with different ratios of cricket flour, resulting in a significant increase in protein content between the formulation with the highest cricket flour content (20%) and the control. Also in literature, cricket flour contains 62% protein, which might be an overestimation due to the presence of the outer covering of insects, the exoskeleton, made of chitin, which contains non-protein nitrogen and can result in the overestimation of the protein [57].

Because edible insects possess essential amino acid profiles that differ from those of conventional meat sources, their incorporation into meat and meat analogue products may favourably augment the amino acids already present in the base formulation. In this regard, edible insect enrichment may improve not only crude protein content but also the amino acid diversity of the final product. The protein-enhancing effect of cricket flour was more evident when it replaced ingredients with little or no protein. Kim et al. [19] reported that replacing 10% fat in meat emulsion with 10% cricket flour resulted in the highest protein content of 20.7%, compared to 14.0% in the control. This increase can be explained by the replacement of back fat, which is a protein-lacking ingredient, with a relatively higher protein-rich cricket flour. The lower moisture content of cricket flour compared to that of pork lean meat and back fat may also have contributed to the higher protein content observed in the meat emulsions. In contrast, when cricket flour was incorporated into texturized vegetable proteins from soy or pea protein concentrates, there was a decrease in protein content. Soy protein concentrate formulations showed reductions of 7% and 9% at 10% and 20% cricket substitution, respectively, while pea protein concentrate formulations of 10% and 20% showed a 3.5% reduction compared with the control [52]. This was due to an increase in random coils and β-turns in the protein–protein interactions and network formation of the cricket flour, contributing to denser structures with reduced porosity [58]. This indicates that insect substitution may have a limited or negative effect when the replaced ingredient is already protein-rich. In the preparation of meat analogue (soy protein) of 5% and 6% cricket powder with (1% or 2%) of rice flour or sticky rice flour and 7% of cricket only through freeze-alignment techniques, cricket powder had a higher soluble protein content of 5.9%, compared to that of soy protein powder of 4.7% [54].

The level of edible insect incorporation influenced the protein outcome. Momchilova et al. [48] incorporated cricket powder at 3.33, 5.0, and 10.0 g/kg^−1^ substitution for soy in raw pork product and observed no significant difference in the protein content between the experimental formulations and the control. This may be due to the relatively low concentration of the cricket powder used, which may not have been enough to cause a noticeable change in the overall protein content. Since both soy and cricket powder are rich sources of protein, replacing soy with cricket powder may have maintained rather than increased the protein levels [59] in the formulations as compared to the control. However, sausages formulated with 2% cricket flour or 2% yellow mealworm recorded higher protein contents of 18.99% and 18.40%, respectively, compared to 16.81%, which corresponds to that of the control [43]. Mealworm-based formulations also demonstrated protein-enhancing potential. Across the reviewed products, protein content ranged from 16.97–48.9% in the experimental formulations and 14.87–34.9% in the controls. The protein content of *Tenebrio molitor* has been verified to be 38%, while that of *Alphitobius diaperinus* has been reported as 61% [18].

Carvalho et al. [41] reported that hybrid hams formulated with 10% *Tenebrio molitor* recorded a substantial increase of 48.9% in protein content, while those formulated with 10% *Alphitobius diaperinus* recorded 46.3% compared with 34.9% in the control. When 5% of *Tenebrio molitor* was combined with 5% of *Alphitobius diaperinus*, the protein content was 40.4%. Although this combined formulation was still higher than the control, it was lower than the individual 10% insect formulations, possibly due to structural modifications or physicochemical interactions among the edible insects used. Similar nonlinear deviations have been reported in an insect snack formulated with *Tenebrio molitor* and *Alphitobius diaperinus*, where the changes were attributed to protein interaction, structural modification, or rearrangement [60]. A concentration-dependent increase was observed in some of the mealworm-enriched products. For instance, the dry fermented sausage formulated with 0, 5, 10, and 15% mealworm flour showed increasing protein content on a dry matter basis as edible insect substitution increased [42]. Consistent with the findings of the present review, a study outside the review corpus reported that replacing pork meat with mealworm flour in frankfurters resulted in a progressive increase in protein content [61]. Some studies have shown that the processing method also influenced protein content. Kim et al. [18] incorporated untreated, defatted, and defatted acid-hydrolysed mealworm larvae into sausage and reported protein contents of 26.08, 30.73, and 31.26 g/100 g, respectively, with the defatted acid-hydrolysed giving the highest protein. Similarly, bologna-type sausage formulated with defatted *Tenebrio molitor* had higher protein contents of 18.55 and 22.52 g/100 g compared with whole *Tenebrio molitor* sausages, which recorded 16.97 and 19.83 g/100 g [37]. In burger analogue formulations made from soybean chop and soybean isolate, protein content decreased from 20.17% in the control to 18.41% and 17.99% as whole buffalo powder increased from 5% to 10%, while lesser mealworm substitution was kept constant at 21.6% [53].

The total protein content of meat or meat analogues formulated with grasshopper ranged from 11.96–51.64%, compared to 10.35–47.66% in their controls. Cruz-López, Álvarez-Cisneros, et al. [51] reported that sausages formulated with 0%, 3%, 5%, 7%, and 10% grasshopper flour replacing potato starch showed increasing protein content as the substitution level increased. Compared to the control, which had 10.35% protein, the formulation with 10% grasshopper flour had 15.37% protein. Similarly, meat analogues formulated with red beans, elephant foot yam, and Javanese grasshopper showed a progressive increase in protein content from 47.66% in the control to 51.24%, 51.62%, and 56.43% at 10%, 20%, and 30% grasshopper inclusion, respectively [55]. These findings support the view that edible insects are more likely to improve protein content when they replace a protein-lacking ingredient. The protein contents observed in this review were within the expected range reported in the literature, as edible insects have been recorded to contain 13–77% protein depending on species, development stage, diet, habitat, gender, season, method, and analytical technique used [62,63]. From the results of this review, the observed protein contents were within the expected range reported in the literature. In instances where there was a protein increase, the edible insect impacted variations in amino acid profile, resulting in products with increased nutrient diversity, as the amino acid profile of edible insects is generally different from that of meat sources, which in turn is dependent on the feed fed to the edible insects [46].

### 4.3. Effect of Edible Insect Enrichment of Meat or Meat Analogue on Dietary Fiber Content

Insect chitin is considered an insoluble, indigestible polysaccharide and functions as a dietary fiber and may benefit the gut microbiome [17,26]. Generally, studies reported an increase in dietary fiber content compared to their controls. Formulations with cricket recorded fiber contents of 8.83–9.42%, while those with mealworm ranged from 0–1.04%. Upon the incorporation of 0%, 10%, and 20% of cricket meal into the texturized vegetable proteins (soy flour, soy protein concentrate, and pea protein concentrate), an increase in insoluble dietary fiber across all formulations was observed [52]. An increase in soluble dietary fiber may be expected when processing treatments that are capable of disrupting insoluble dietary fiber into smaller soluble fragments are applied, as observed in previous studies [64,65]. In this study, the soluble dietary fiber remained constant across all formulations. Apart from the soy flour texturized vegetable protein, which consistently had high total dietary fiber, the addition of cricket meal resulted in no significant changes [52]. When the cricket flour was replaced at an increased level, the acid detergent fiber content of the meat emulsion was increased (*p* = 0.0124), which is approximately 8.5% of the dry matter of the chitin from the exoskeleton [19]. Momchilova et al. [48] found that the high levels of dietary fiber in experimental samples compared to the control can be due to the chitin contained in the cricket powder.

The dry fermented sausage formulated with 0%, 5%, 10%, and 15% mealworm flour showed an increase in dry matter dietary fiber as the edible insect substitution increased. This significant increase in the total fiber was attributed to the 3.3 g/100 g of fiber in the mealworm and the absence of fiber in the meat [42]. The whole and defatted *Tenebrio* powder used as a partial replacement of fat at 7.5% and 15% each showed an increase in total fiber content with increasing powder concentration. With a total dietary fiber of 0.41 and 0.83 g/100 g recorded for the 7.5% and 15% of the defatted powder and 0.25 and 0.49 g/100 g for the 7.5% and 15% replacement of the whole powder, respectively. There was no total dietary fiber present in the control for the bologna-type sausage formulated [37].

### 4.4. Effect of Edible Insect Powder Enrichment of Meat or Meat Analogue on Fat Content

There was a general reduction in fat content following edible insect enrichment, but this was different for products enriched with edible insect larvae. Edible insect larvae are typically high in fats, with fat content ranging from 24.70–43.08% per 100 g for *Tenebrio molitor* larvae and 52.4–60.1% for the larvae from palm weevils [27,66]. Fat content in cricket-based and mealworm-based formulations ranged from 0.6–9.18% and 5.04–51.95%, respectively, compared to the control at 0.20–14.93% and 15.49–52.93%. In instances where larvae were used for enrichment, there was an improved fatty acid profile [39,67].

The fat content of beef burger formulated with 0%, 8.25%, 12.5%, and 25% of cricket flour showed a higher fat content of 9.18% in the formulation with 25% and lower fat content in the formulation with 8.25% [21]. Zafar et al. [68] reported 21.8% lipid content for *Gryllus assimilis*, which is one of the cricket species with a very high amount of lipid. It also has a high amount of linoleic acid, known to have numerous health benefits [69,70]. The texturized vegetable protein of soy flour, soy protein, and pea protein concentrate formulated with 0%, 10%, and 20% cricket meal each resulted in a fat content increase across all three matrices, with 3.5, 1.3, and 3.3 g/100 g reported to their respective 20% formulation [52]. Cooked sausage formulated with 2% cricket flour resulted in a fat content of 14.48% compared to the control, which had 12.28% [43].

Momchilova et al. [48] reported that there was no significant difference between the fat content of the control and the experimental sample that incorporated 3.33, 5, and 10 g/kg^−1^ of cricket flour into the raw meat model. The hybrid ham formulated with 10% *Tenebrio molitor* was reported to contain 40.1% fat content compared to that of the control, which was 24.2%, but when 5% of the *Tenebrio molitor* was incorporated with 5% *Alphitobius diaperinus*, the fat content decreased to 36.8% [41]. The fat content (dry matter) of 0%, 5%, 10%, and 15% of mealworm flour incorporated into dry fermented sausage showed a decrease in fat levels with increasing substitution [42]. This finding has been observed in emulsion sausage that was formulated with the same ingredient used for the dry fermented sausage [61]. The incorporation of 7.5% and 15% of either whole or defatted mealworm powder into bologna-type sausage resulted in the reduction of its fat content; compared to the control sausage at 15.49%, the fat content decreased to 6.65% and 5.04% for the defatted formulations and to 9.45% and 8.44% for the whole mealworm flour formulations [37]. Whole buffalo powder of 0%, 5%, and 10% incorporated into a burger analogue of soy chop and soy protein isolate with 0% and a constant 21.6% of lesser mealworm was observed to increase total lipid content with increasing whole buffalo powder content [53].

### 4.5. Effect of Edible Insect Powder Enrichment of Meat or Meat Analogue on Ash Content

Some studies reported no significant change in ash content between experimental samples and their controls [19,37,42]. Albuquerque et al. [21] formulated beef burgers with 0%, 8.25%, 12.50%, and 25% cricket powder, with the 25% formulation having the highest ash content of 5.61% compared to the control of 3.56% and 4.44%, 4.91%, and 5.06% for the other formulations, respectively. The ash content decreased with an increasing incorporation of cricket meal into soy flour texturized vegetable protein, ranging between 4.6–6.9 g/100 g (d.m) and 8.9–10.3 g/100 g (d.m) for soy protein concentrate texturized vegetable protein and no significant change for that of pea protein concentrate texturized vegetable protein [52]. Cooked sausage formulated with 2% cricket powder had an ash content of 1.30% compared to 0.70% of the control and 1% for soy protein [43]. The raw meat model formulated with spirulina and cricket powder recorded the highest ash content of 3.54% [48]. The ash content of 10% *Tenebrio molitor* incorporated into a hybrid ham was 9.6% compared to 7.8% for the control and 9.6% when 5% of the *Tenebrio molitor* was incorporated with 5% *Alphitobius diaperinus* [41]. Bologna-type sausage formulated with 7.5% and 15% defatted and whole *Tenebrio* powder resulted in higher ash content than the control, with 3.13 g/100 g being the highest for 15% whole *Tenebrio* powder [37].

Grasshopper flour incorporated into sausage at 3%, 5%, 7%, and 10% had ash content of 3.21%, 3.19%, 3.05%, and 3.41%, respectively, compared to 2.53% for the control [51]. The ash content of the meat analogue formulated with Javanese grasshopper increased with increasing substitution of 0%, 10%, 20%, and 30%. The control had an ash content of 5.37% compared with 5.62%, 5.77%, and 6.38%, respectively, for the formulations with increasing grasshopper amount [55]. The ash content of grasshopper has been found to be 1.46%, which would have the potential to increase ash content when used in higher concentrations [71]. High levels of calcium, magnesium, potassium, sodium, and phosphorus were recorded in some formulations incorporating mealworm into meat or meat analogues [37,41]. The incorporation of 10% cricket powder into a meat emulsion formulation resulted in increased levels of minerals such as magnesium, potassium, phosphorus, zinc, and manganese [19].

### 4.6. Effects of Edible Insect Enrichment on the Techno-Functional, Textural, and Sensory Attributes of Meat and Meat Analogues

Pre-treatment methods of edible insect ingredients were identified as key determinants of the textural properties of insect-enriched meat and meat analogues. Processes such as defatting, drying (oven, microwave, and freeze-drying), hydrolysis, and protein extraction significantly influenced textural outcomes. Defatting generally increased hardness and chewiness by removing lipids that act as plasticisers, thereby enhancing protein–protein interactions and matrix rigidity [18,37]. Drying methods also play a critical role, with microwave and oven drying producing firmer and denser textures due to greater protein denaturation, whereas freeze-drying better preserved protein structure and was associated with softer textures [38]. Additionally, advanced treatments such as ultrasonication and alkaline extraction modified protein structure and solubility, thereby influencing functional properties including firmness, adhesiveness, and water-binding capacity [35,44]. Overall, these findings highlight pre-treatment as a crucial factor in tailoring the functional performance of insect-based ingredients in food systems.

The textural attributes of food materials are essential as they impact the sensory properties of the resulting food products. Foods with improved textural attributes impact the overall acceptable mouthfeel experience for consumers [72]. The textural attributes objectively include the hardness, springiness, cohesiveness, and gumminess [72]. For meat and meat analogue products, their structural presentation, determined by their textural composition, is technologically important as it can affect their cooking properties, especially the cooking losses [73]. Food products with higher cooking losses may present defects in their textural composition [73]. This is a major problem, particularly for meat analogues, especially when formulation ingredients are depleted of proteins and polysaccharides associated with structure formation. The use of extrusion technology has become a common practice in meat analogue production as a strategy to overcome that barrier [74]. The high-pressure and shearing process allows for a complete mixing of the ingredients [74]. Another common approach has been the use of certain food hydrocolloids, usually polysaccharides such as xanthan gum and guar gum, which act as binding agents in meat analogues [75].

The findings from this review (Table 2) demonstrate that edible insect enrichment generally enhances key textural strength parameters, particularly hardness, chewiness, and gumminess. Several studies reported significant increases in these attributes with increasing levels of insect incorporation in products such as beef burgers, sausages, and meat emulsions [19,21,76]. This trend can be attributed to the high protein content and functional properties of insect proteins, which promote gel formation and increase matrix density. Similarly, improvements in cohesiveness and structural strength were observed in some formulations, further supporting the role of insect proteins in reinforcing product structure [34,46]. However, these improvements are not universally beneficial. A study reported reductions in springiness and cohesiveness, particularly at higher substitution levels, indicating a trade-off between firmness and elasticity [54]. This suggests that while insect proteins enhance structural rigidity, they may reduce the elastic recovery of the product, potentially negatively affecting mouthfeel in certain applications.

The impact of insect enrichment on water holding capacity (WHC) was inconsistent across studies. While some formulations exhibited improved WHC due to enhanced protein–water interactions and network formation [34], others showed reductions, particularly in highly processed or extruded systems [46,50]. These variations highlight the importance of processing techniques such as drying, defatting, hydrolysis, and extrusion in modulating protein functionality and water retention properties. Notably, improvements in structural integrity were also associated with reduced cooking losses in some cases, indicating enhanced technological performance of the enriched products [39].

Overall, the acceptable substitution levels for maintaining desirable textural properties tend to be relatively low in conventional meat products, typically ranging between 2.5% and 12.5%, beyond which excessive hardness and reduced elasticity may compromise product quality. In contrast, higher inclusion levels are more feasible in meat analogue systems, where structural expectations differ, and formulation strategies (e.g., extrusion and hydrocolloid addition) can be optimized to accommodate higher insect incorporation. Importantly, different age groups may vary in their preferences for the textural properties of food. Older adults, for example, may prefer food products that are generally easier to chew and swallow due to factors such as loss of teeth and dysphagia, which remain common age-related challenges [77]. Younger adults may also prefer relatively harder and chewier foods. Consequently, attention to the consumer profile and preference characteristics should be taken into consideration during the reformulation of meat and meat analogues containing edible insects.

The sensory properties of newly developed foods are essential as they influence consumer acceptance of these products [78]. These properties include the appearance, taste, mouthfeel, aftertaste, and overall liking of the products [78]. Consumer surveys investigating preferences for purchasing meat analogues versus meat-based products show that preferences for meat analogues are generally lower due to factors such as poor taste and texture [5]. Enrichment of meat analogues with edible insects could be a strategy to improve their sensory properties [79]. In this review, enrichment of meat and meat analogues increased the liking of sensory attributes of the products compared to the control. In most cases, substitution levels of up to 10% were associated with improved overall liking compared to control samples. Several studies have investigated the incorporation of edible insect protein into meat products as a strategy to improve nutritional quality while maintaining acceptable sensory properties.

Albuquerque et al. [21] demonstrated that the incorporation of cricket flour and soy protein significantly increased the protein content of beef burgers. The response surface analysis showed significant effects on hardness, springiness, cohesiveness, lightness, and yellowness. Among the formulations tested, C12S0 was considered the most suitable because it caused minimal alterations in texture and colour difference that were not perceptible to consumers. The study also highlighted the importance of balancing pro-oxidant and antioxidant activities in insect-enriched products. Similarly, Choi et al. [34] investigated edible insect powder as a partial meat substitute in pork patties and reported improvements in physicochemical properties, such as increased protein, carbohydrate, fat, ash content, pH, and water holding capacity, alongside reduced cooking loss by employing 10 trained panelists. Texture profile analysis revealed increased hardness, chewiness, and gumminess compared to controls. Overall consumer preference scores for pork patties containing 20% edible insect powder compared with control samples were lower, indicating that sensory acceptability remains a major challenge despite functional improvements.

The findings by Cruz-López, Escalona-Buendía, et al. [35] showed that alkalization-piezoelectric ultrasound treatment improved the solubility and techno-functional properties of grasshopper protein when incorporated into sausages using 100 consumers. Importantly, sausages containing 5% meat substitution exhibited sensory acceptability comparable to the control, whereas acceptance declined significantly at 10% inclusion, suggesting that low inclusion levels may successfully maintain consumer acceptance. Likewise, Kim et al. [18] reported that defatting and acid hydrolysis increased the protein content of insect flours, although reductions in pH, redness, yellowness, and protein solubility were observed. Despite these changes, the treated flours did not adversely affect colour, protein solubility, or cooking yield in emulsion sausages, indicating their suitability as functional ingredients in processed meat products. Also, Park et al. [36] found that incorporating 15% silkworm pupae improved pH, viscosity, hardness, gumminess, and chewiness of meat batter formulations. The addition of transglutaminase further enhanced these physicochemical properties, suggesting that silkworm pupae may serve as both a nutritional and functional ingredient in meat systems.

Sensory quality remains one of the primary limitations of insect incorporation at high substitution levels. Rodríguez-Párraga et al. [37] reported that sausages formulated with defatted insect powder received higher acceptance scores than those containing whole powder because defatting appeared to reduce unpleasant odours. Using 50 consumers for the sensory evaluation, it was observed that all reformulated sausages, except those containing 15% whole mealworm powder, maintained overall acceptance scores above the neutral point. Replacing 15% of pork fat with whole *Tenebrio molitor* powder significantly reduced overall sensory acceptability. Similarly, Zhang et al. [38] studies on hybrid frankfurters consistently demonstrated declining sensory and structural quality with increasing insect substitution using 16 panelists. The study comparing freeze-dried and microwave-dried yellow mealworm larvae flour showed that microwave-dried flour improved texture, gel properties, and rheological behaviour more effectively than freeze-dried flour. However, increasing substitution ratios reduced sensory scores compared with standard frankfurters, although 10% microwave-dried mealworm flour was identified as the optimal replacement level. Consistent with these findings, Zhang et al. [40] reported that incorporating *Tenebrio molitor* larvae protein reduced fat content and increased ash content while maintaining cooking loss and sensory properties comparable to the control at 5–15% inclusion levels. However, insect incorporation weakened gel network formation, resulting in softer sausages with poorer textural properties despite improved digestibility.

Further evidence was provided by Hospital et al. [42], who observed that replacing pork meat with mealworm flour significantly increased protein, fiber, and polyunsaturated fatty acid contents while reducing lipid oxidation. However, higher substitution levels caused darker colour development and lower sensory acceptability. Sensory evaluation using 18 panelists indicated that products containing up to 5% replacement maintained acceptable quality, whereas higher levels caused significant sensory deviations. Comparable outcomes were observed by Kim et al. [19], who reported that replacing lean meat and fat with house cricket flour at levels up to 10% enhanced protein and micronutrient contents without negatively affecting cooking yield or textural properties. In contrast, Kolev et al. [43] found that insect protein addition negatively affected all evaluated sensory attributes, with sausages containing yellow mealworm flour receiving particularly low scores for appearance, texture, and flavour. Similarly, Cruz-López, Álvarez-Cisneros, et al. [51] reported increased hardness, springiness, gumminess, and chewiness following grasshopper flour incorporation, alongside reduced lightness. Sensory analysis using 100 consumers aged 19–40 years showed that sausages containing 5% grasshopper protein extract achieved liking scores comparable to the control, whereas 7–10% grasshopper flour was associated with herbal flavour, brown colour, and granular texture, resulting in lower consumer liking.

Storage stability and antioxidant potential have also been highlighted in several studies. Singh et al. [44] reported improved sensory quality and preservative effects in meat emulsions enriched with locust protein hydrolysates, likely due to delayed oxidative and microbial spoilage during storage. Similarly, Yong et al. [45] demonstrated that adding edible insect extracts, particularly 2% *Allomyrina* dichotoma, reduced lipid oxidation and maintained microbial quality without negatively affecting product quality. Finally, Scholliers et al. [39] observed that replacing even 5–10% of meat with insect protein substantially reduced structural and textural properties. Although higher heating temperatures improved viscoelastic characteristics in some formulations, textural quality remained inferior to that of conventional sausages.

Boonarsa et al. [46] demonstrated that soy-wheat (SW)-based meat analogues exhibit concentration-dependent improvements in apparent viscosity, texture, amino acid composition, and essential amino acid index (EAAI), with higher SW levels enhancing firmness, cohesiveness, and chewiness, while lower levels (5–10%) promote water holding capacity and fiber-like structure formation. Similarly, Kim et al. [47] reported that blending textured vegetable protein (TVP) with edible insect protein (EIP) in restructured jerky systems modifies thermal stability and texture, with a 60:40 TVP–EIP ratio yielding optimal sensory and structural properties, while increasing insect inclusion enhances EAAI. Complementing this, Kim et al. [49] highlighted that protein–protein interactions, particularly when enhanced through transglutaminase treatment, improve emulsion stability and physical properties, although optimal quality requires balanced protein blending rather than single-source formulations.

In insect-enriched systems, Carvalho et al. [41] assessed hybrid hams using 40 untrained consumers and found that hybrid ham products containing 5–10% insect powders (*Tenebrio molitor* and *Alphitobius diaperinus*) were most acceptable sensorially, with increased firmness contributing positively to texture perception, while Chao et al. [50] showed that mealworm incorporation (15–30%) combined with higher extrusion temperatures (180 °C) promotes fibrous structure formation through enhanced disulfide bonding and protein crosslinking. Similarly, Krawczyk et al. [53] reported optimal consumer acceptability at low inclusion levels (5%) of alternative protein powders, reinforcing the importance of controlled substitution levels using 24 panelists. Nakagawa et al. [54] further demonstrated that cricket protein exhibits superior soluble protein content relative to soy, enhancing fiber-like gel formation, firmness, and adhesion, particularly when combined with carbohydrate matrices such as sticky rice flour. Priyatnasari et al. [55] confirmed the nutritional potential of insect-based formulations, with grasshopper and kidney bean combinations yielding high-protein, low-cholesterol meat analogue patties, while Bottle et al. [52] showed that 10% cassava meal inclusion improves techno-functional properties such as water and oil absorption, while at 20% incorporation, it enhances protein digestibility.

Across the included studies, acceptable levels of edible insect incorporation vary markedly depending on the food matrix and the extent to which product structure is derived from native meat proteins versus engineered formulation systems. In conventional meat-based products such as burgers, sausages, frankfurters, and emulsified meat systems, edible insect substitution is generally constrained to low levels. Most studies indicate that approximately 2.5–10% inclusion is associated with optimal or acceptable sensory quality, with limited cases extending to about 15% under optimized processing conditions. Beyond this range, consistent deterioration in sensory attributes—particularly increased hardness, reduced springiness, darkening of colour, and emergence of off-flavours—tends to outweigh functional benefits. This limitation reflects the sensitivity of intact or semi-restructured meat matrices, in which small compositional changes can significantly disrupt muscle fiber integrity, fat distribution, and collagen-based textural behaviour.

In contrast, higher substitution levels are consistently achievable in meat analogue systems, including plant–insect hybrids, textured vegetable protein (TVP) blends, extrusion-based products, jerky analogues, and other restructured formulations. In these systems, acceptable inclusion levels typically range from approximately 10% to 40%, with some studies reporting functional performance and acceptable sensory outcomes at levels approaching 50–60%, depending on formulation strategy and processing conditions. The greater tolerance in these systems is attributable to their engineered structural architecture, in which texture is developed through protein–protein interactions, extrusion-induced fiber alignment, and the use of structuring agents such as hydrocolloids or enzymatic crosslinkers, rather than relying on native meat structure.

### 4.7. Quality Assessment of Included Studies

The quality appraisal demonstrated that the available evidence is generally supported by studies of high methodological quality. Most investigations clearly stated their objectives, employed appropriate experimental designs, used recognized analytical methods, and adequately reported statistical analyses. Furthermore, formulation procedures and outcome measures were generally described in sufficient detail to permit interpretation and comparison across studies. Nevertheless, some studies provided limited information about replication procedures, which may affect the reproducibility and precision of reported findings. Although these limitations do not substantially weaken the overall conclusions of the review, they highlight the need for greater methodological standardization and more comprehensive reporting in future edible insect food product development studies.

### 4.8. Limitations

This systematic review has several limitations that should be considered when interpreting the findings. First, substantial heterogeneity existed among the included studies regarding edible insect species, processing methods, substitution levels, product formulations, and analytical approaches. The reviewed studies investigated different insect species, including mealworm, cricket, grasshopper, silkworm pupae, and superworm, which differ considerably in their nutritional composition and techno-functional properties. Consequently, direct comparison of outcomes across studies should be interpreted with caution. The studies also evaluated a wide range of food matrices, including sausages, burgers, patties, meat emulsions, frankfurters, hams, and meat analogue products. These products differ substantially in their formulation characteristics and processing conditions, which may influence how edible insect incorporation affects nutritional composition, physicochemical properties, and consumer acceptability. Therefore, the findings may not be universally applicable across all meat-based and meat analogue systems.

Second, considerable variation was observed in the methods used to assess nutritional composition, techno-functional properties, and sensory acceptability. Differences in analytical methods, reporting units, sensory panel characteristics, and evaluation protocols limited direct comparisons among studies and prevented quantitative meta-analysis. As a result, the evidence synthesis was primarily qualitative. Some studies reported results on a dry matter basis while others reported values on a wet weight basis, which may have contributed to variability in reported nutrient concentrations. Additionally, several studies did not consistently report measures of variability, sample size justification, or statistical significance for all outcomes evaluated.

A further limitation relates to the measurement of protein content in insect-enriched products. Most of the included studies determined protein using nitrogen-based methods such as the Kjeldahl or Dumas procedures. These methods estimate protein from total nitrogen content and may overestimate true protein levels in edible insect ingredients because chitin, a structural polysaccharide present in the insect exoskeleton, contains non-protein nitrogen. Since most studies did not apply chitin-specific nitrogen correction factors or directly quantify true protein content, some of the increases in protein reported in Table 1 may reflect contributions from chitin-derived nitrogen rather than solely increases in digestible protein. Consequently, the magnitude of protein enhancement observed across studies should be interpreted with caution.

Under the European Union Novel Foods Regulation (EU) 2015/2283 [80], insect-based foods are subjected to pre-market authorization, and only approved species may be legally marketed as food within the EU. At present, regulatory approval is species-specific and not universal across edible insects. Among the species included in this review, *Acheta domesticus* (house cricket) has received EU novel food authorization for food use; however, *Gryllus bimaculatus* (field cricket) and *Protaetia brevitarsis seulensis* (white-spotted flower chafer) are not currently authorized as novel foods within the European Union. Consequently, while evidence on their nutritional and functional properties is included for scientific completeness, their commercial application within the EU remains restricted. Regulatory status in other jurisdictions may differ; therefore, findings should be interpreted within the context of region-specific food safety frameworks.

Finally, the review was restricted to studies published in English and indexed within PubMed, Scopus, and ScienceDirect. Although these databases provide extensive coverage of food science, nutrition, and health-related research, relevant studies indexed exclusively in other databases, such as Web of Science, may not have been identified despite the comprehensive search strategy employed. This language restriction may have introduced potential language bias, particularly by excluding relevant studies published in Korean and other Asian languages, where research on edible insects is actively conducted. Consequently, the geographical distribution of the included studies may not fully reflect the global evidence base. The review was also not prospectively registered, which should be considered when evaluating the methodological rigor of the review process. The quality appraisal tool used was adapted from a general methodological guide, which may have limited discriminative power and could lead to overestimation of study quality, as all included studies clustered at high scores. Despite these limitations, the review provides a comprehensive synthesis of current evidence on the effects of edible insect enrichment on the nutritional composition, techno-functional properties, and consumer acceptability of meat-based and meat analogue products and identifies important areas for future research.

### 4.9. Future Perspective and Directions

Despite the promising potential of edible insect enrichment for improving the nutritional and functional properties of meat-based and meat analogue products, several research gaps remain. Future studies should prioritize the development of standardized formulation, processing, and sensory evaluation protocols to improve comparability across studies and facilitate evidence synthesis. Greater attention should also be given to the application of analytical methods that distinguish true protein from non-protein nitrogen sources, particularly chitin-derived nitrogen, to provide more accurate estimates of protein quality and nutritional value.

Further investigations are required to evaluate the allergenicity and food safety assessment related to edible insect enrichment. This is particularly relevant for commonly used species such as *Tenebrio molitor* and *Acheta domesticus*, which are known to exhibit cross-reactivity with crustacean allergens due to shared proteins, including tropomyosin and arginine kinase. None of the studies evaluated allergenic risk or reported safety outcomes, despite potential regulatory implications under labelling frameworks such as EU Regulation 1169/2011. Future research should incorporate allergenicity testing and food safety evaluation of all insect-based ingredients as standard components of insect-enriched food development to ensure consumer protection and regulatory compliance.

Long-term storage studies are also needed to assess the effects of edible insect incorporation on product stability, shelf life, lipid oxidation, microbial quality, and consumer acceptability during storage. In addition, future research should move beyond product formulation and characterization to include well-designed human studies that evaluate the nutritional, physiological, and health impacts of regular consumption of insect-enriched meat and meat analogue products. Addressing these research priorities will contribute to the development of safe, nutritionally beneficial, and consumer-acceptable edible insect-based food systems.

## 5. Conclusions

This systematic review has provided a comprehensive assessment of the effects of edible insect enrichment on meat-based products and meat analogues, with particular emphasis on their nutritional composition, techno-functional properties, and consumer acceptability. The findings from the reviewed studies clearly demonstrate that edible insects possess significant potential as alternative protein sources and functional ingredients in food product development. From a nutritional perspective, incorporating edible insect powders consistently enhanced the protein content, dietary fiber, and mineral composition of both meat and meat analogue products. These improvements contribute to increased nutrient density and dietary quality of the enriched products. In addition, the enrichment process improved the amino acid profile, particularly in plant-based meat analogues, thereby addressing one of the major limitations of plant proteins: their incomplete essential amino acid composition.

Regarding techno-functional properties, the inclusion of edible insect powders generally improved the structural characteristics of the products. Increases in hardness, chewiness, and cohesiveness were commonly reported, indicating enhanced protein network formation and structural stability. These improvements are particularly beneficial in meat analogue systems, where achieving a meat-like texture remains a major challenge. In terms of sensory properties and consumer acceptability, the findings indicate that edible insect enrichment can improve flavour, texture, and overall acceptability when used at moderate levels. The evidence demonstrates that the incorporation of edible insects does not conform to a single optimal substitution threshold. Instead, acceptable inclusion levels are strongly matrix-dependent. Conventional meat systems exhibit a narrow tolerance window centered at low inclusion levels, whereas meat analogue systems permit substantially higher without compromising structural integrity. This distinction is critical for interpreting techno-functional and sensory outcomes and underscores the need to report substitution levels by product type rather than as a universal benchmark.

Overall, edible insect enrichment represents a promising strategy for enhancing the nutritional and functional properties of meat and meat analogue products. However, achieving optimal product quality and consumer acceptance requires careful consideration of insect species, processing methods, and substitution levels. Future advancements in food processing technologies and increased consumer education will be essential to support the large-scale adoption of insect-based food products. Also, studies including well-designed human intervention trials are needed to determine whether the observed compositional improvements translate into meaningful health outcomes in human populations.

## Figures and Tables

**Table 3:** Quality appraisal and risk-of-bias assessment of studies evaluating edible insect enrichment in meat and meat analogue products.

Authors, Year	Aims	Study Design	Methods	Correct Measurement Tools	Formulations	Replication	Outcome Reporting	Statistical Analysis	Conclusions Supported	Score
Albuquerque et al. [21]	Yes	Yes	Yes	Yes	Yes	Yes	No	Yes	Yes	8/9 (high)
Choi et al. [34]	Yes	Yes	Yes	Yes	Yes	Yes	No	Yes	Yes	8/9 (high)
Cruz-López, Escalona-Buendía, et al. [35]	Yes	Yes	Yes	Yes	Yes	Yes	Yes	Yes	Yes	9/9 (high)
Kim et al. [18]	Yes	Yes	Yes	Yes	Yes	No	Yes	Yes	No	7/9 (high)
Park et al. [36]	Yes	Yes	Yes	Yes	Yes	Yes	No	Yes	Yes	8/9 (high)
Rodríguez-Párraga et al. [37]	Yes	Yes	Yes	Yes	Yes	Yes	Yes	Yes	Yes	9/9 (high)
Zhang et al. [38]	Yes	Yes	Yes	Yes	Yes	Yes	Yes	Yes	No	8/9 (high)
Scholliers et al. [39]	Yes	Yes	Yes	Yes	Yes	Yes	No	Yes	Yes	8/9 (high)
Zhang et al. [40]	Yes	Yes	Yes	Yes	Yes	Yes	Yes	Yes	No	8/9 (high)
Kim et al. [19]	Yes	Yes	Yes	Yes	Yes	No	No	Yes	Yes	7/9 (high)
Carvalho et al. [41]	Yes	Yes	Yes	Yes	Yes	No	Yes	Yes	Yes	8/9 (high)
Hospital et al. [42]	Yes	Yes	Yes	Yes	Yes	No	Yes	Yes	Yes	8/9 (high)
Kolev et al. [43]	Yes	Yes	Yes	Yes	Yes	No	Yes	Yes	Yes	8/9 (high)
Singh et al. [44]	Yes	Yes	Yes	Yes	Yes	Yes	Yes	Yes	Yes	9/9 (high)
Yong et al. [45]	Yes	Yes	Yes	Yes	Yes	Yes	Yes	Yes	No	8/9 (high)
Boonarsa et al. [46]	Yes	Yes	Yes	Yes	Yes	Yes	Yes	Yes	Yes	9/9 (high)
Kim et al. [47]	Yes	Yes	Yes	Yes	Yes	Yes	No	Yes	Yes	8/9 (high)
Momchilova et al. [48]	Yes	Yes	Yes	Yes	Yes	No	Yes	Yes	Yes	8/9 (high)
Kim et al. [49]	Yes	Yes	Yes	Yes	Yes	Yes	No	Yes	Yes	8/9 (high)
Chao et al. [50]	Yes	Yes	Yes	Yes	Yes	No	Yes	Yes	Yes	8/9 (high)
Cruz-López, Álvarez-Cisneros, et al. [51]	Yes	Yes	Yes	Yes	Yes	No	Yes	Yes	Yes	8/9 (high)
Bottle et al. [52]	Yes	Yes	Yes	Yes	Yes	No	Yes	Yes	Yes	8/9 (high)
Krawczyk et al. [53]	Yes	Yes	Yes	Yes	Yes	No	Yes	Yes	Yes	8/9 (high)
Nakagawa et al. [54]	Yes	Yes	Yes	Yes	Yes	No	Yes	Yes	Yes	8/9 (high)
Priyatnasari et al. [55]	Yes	Yes	Yes	Yes	Yes	No	Yes	Yes	No	7/9 (high)

## Data Availability

No new data were created or analyzed in this study. Data sharing is not applicable to this article.

## Supplementary Materials

The following supporting information can be downloaded at: https://www.mdpi.com/article/10.3390/foods15132329/s1, Table S1: PRISMA 2020 Checklist for Effects of Edible Insect Enrichment of Meat-Based and Meat Analogue Products on the Nutritional Composition, Techno-Functional Properties, and Acceptability to Consumers: A Systematic Review, Table S2: Publication output showing the application of edible insects in meat-based and meat analogue products over 10 years (2015–2025) obtained from PubMed database on 5 May 2026.
